# 10,12-Dimethyl­pteridino[6,7-*f*][1,10]phenanthroline-11,13(10*H*,12*H*)-dione–chloro­form (1/1)

**DOI:** 10.1107/S1600536810031570

**Published:** 2010-08-11

**Authors:** Waynie Olaprath, Jennifer Roden, Kraig A. Wheeler, Mark E. McGuire

**Affiliations:** a Department of Chemistry, Eastern Illinois University, 600 Lincoln Ave., Charleston, IL 61920, USA

## Abstract

In the title co-crystal, C_18_H_12_N_6_O_2_·CHCl_3_, intra­molecular Cl_3_C—H⋯N hydrogen-bonding inter­actions occur between a single CHCl_3_ and both N atoms at the 1,10-positions on the phenanthroline portion of the mol­ecule. The inter­planar distance between inversion-related mol­ecules is 3.241 (2) Å.

## Related literature

For the synthesis, see: Black *et al.* (1993 ▶). For the possible use of metal complexes of this ligand as DNA probes, see: Gao *et al.* (2007 ▶); Lawrence *et al.* (2006 ▶). For studies involving the non-methyl­ated analog of the title compound, see: Chen *et al.* (2010 ▶); Dalton *et al.* (2008 ▶); Ozawa *et al.* (2006 ▶). For a related stucture, see: Ton & Bolte (2005 ▶). For Cl_3_C—H⋯N hydrogen bonding, see: Fan *et al.* (2009 ▶); Li & Wang (2007 ▶).
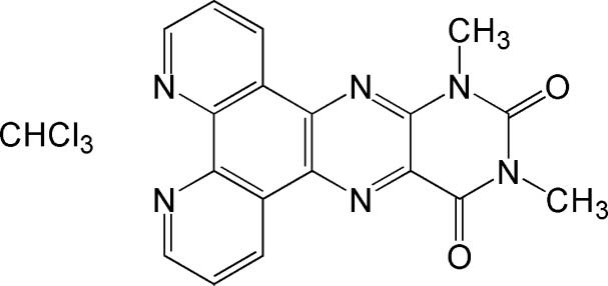


## Experimental

### 

#### Crystal data

C_18_H_12_N_6_O_2_·CHCl_3_
*M* *_r_* = 463.70
Monoclinic, 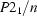

*a* = 8.9043 (2) Å
*b* = 16.4009 (4) Å
*c* = 13.4872 (4) Å
β = 108.058 (1)°
*V* = 1872.63 (8) Å^3^
*Z* = 4
Cu *K*α radiation
μ = 4.72 mm^−1^
*T* = 173 K
0.45 × 0.22 × 0.17 mm

#### Data collection

Bruker APEXII CCD diffractometer
Absorption correction: numerical (*SADABS*; Bruker, 2008 ▶) *T*_min_ = 0.224, *T*_max_ = 0.508
15543 measured reflections
3370 independent reflections
3115 reflections with *I* > 2σ(*I*)
*R*_int_ = 0.033

#### Refinement

*R*[*F* ^2^ > 2σ(*F* ^2^)] = 0.032
*wR*(*F* ^2^) = 0.095
*S* = 1.08
3370 reflections
273 parameters
H-atom parameters constrained
Δρ_max_ = 0.37 e Å^−3^
Δρ_min_ = −0.26 e Å^−3^

### 

Data collection: *APEX2* (Bruker, 2008 ▶); cell refinement: *APEX2* and *SAINT* (Bruker, 2008 ▶); data reduction: *SAINT* and *XPREP* (Bruker, 2008 ▶); program(s) used to solve structure: *SHELXS97* (Sheldrick, 2008 ▶); program(s) used to refine structure: *SHELXL97* (Sheldrick, 2008 ▶); molecular graphics: *X-SEED* (Barbour, 2001 ▶); software used to prepare material for publication: *X-SEED*.

## Supplementary Material

Crystal structure: contains datablocks I, New_Global_Publ_Block. DOI: 10.1107/S1600536810031570/fl2311sup1.cif

Structure factors: contains datablocks I. DOI: 10.1107/S1600536810031570/fl2311Isup2.hkl

Additional supplementary materials:  crystallographic information; 3D view; checkCIF report

## Figures and Tables

**Table 1:** Hydrogen-bond geometry (Å, °)

*D*—H⋯*A*	*D*—H	H⋯*A*	*D*⋯*A*	*D*—H⋯*A*
C19—H19⋯N1	1.00	2.39	3.188 (2)	136
C19—H19⋯N6	1.00	2.26	3.181 (2)	152

**Fig. 1.**
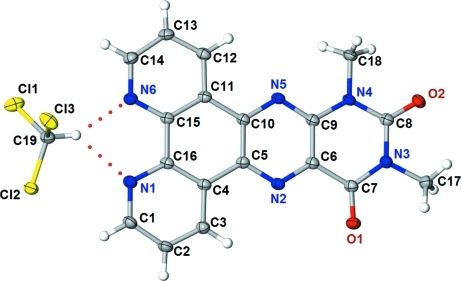
Perspective view of the title compound, with the atom numbering; displacement ellipsoids are at the 50% probability level.

**Fig. 2.**
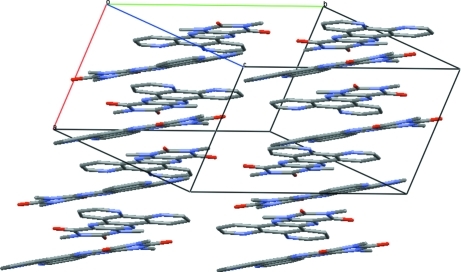
Packing diagram for the title compound. The molecular planes of alternating stacks of molecules are oriented at 12.46 (2)° relative to one another. The CHCl3 molecules and H-atoms have been omitted for clarity.

## supplementary crystallographic information

### Comment

We first reported the synthesis of the title compound as part of our effort to study the pH-dependent electron transfer reactions of transition metal complexes of riboflavin mimics (Black *et al.*, 1993). Since that time, others have investigated metal complexes of this ligand as possible DNA probes (see, for example, Gao *et al.*, 2007; Lawrence *et al.*, 2006). The title compound is only slightly soluble in many common solvents; it appears at least moderately soluble in CHCl_3_. We have grown crystals by vapor diffusion of hexane into a CHCl_3_/95% EtOH solution. As shown in Figure 1, the crystal structure revealed interesting H-bonding interactions involving the H-atom on CHCl_3_ and the N-atoms in the 1,10-phenanthroline portion of the ligand (C19—H···N1: 3.188 (2) Å, 136° and C19—H···N6: 3.181 (2) Å, 152°). This type of interaction has been reported previously (Ton & Bolte, 2005) for a co-crystal of CHCl_3_ and 1,10-phenanthroline(Cl_3_C—H···N: 3.175 (3) Å, 146° and 3.225 (3) Å, 141°). *Ab initio* calculations on the interaction between CHCl_3_ and pyridine (Li & Wang, 2007) predict a range of Cl_3_C—H···N distances (3.13–3.25 Å) and angles (156–167°), depending on the basis sets used. Fan *et al.* (2009) reported very weak Cl_3_C—H···N interaction in pyridine/chloroform solutions as measured by anti-Stokes Raman scattering. The interplanar distance between inversion-related molecules in I (viewed along the *a*-axis) is 3.241 (2) Å. The molecular planes in alternating stacks are oriented at 12.46 (2)° to each other; this could at least partially be due to packing constraints imposed by the slightly out-of-plane C18-methyl group (see packing diagram in Figure 2). Studies involving the non-methylated analog of the title compound have also been reported (Chen *et al.*, 2010; Dalton *et al.*, 2008; Gao *et al.*, 2007; Ozawa *et al.*, 2006) although, to our knowledge, no crystal structures of this analog have been reported.

### Experimental

The title compound was prepared and purified using a previously published procedure (Black *et al.*, 1993). A small amount of this compound was dissolved in ~1 ml of CHCl_3_ and one drop of 95% ethanol was added to the mixture. The vial containing this mixture was placed in a beaker with ~1 ml of hexane and the beaker was loosely sealed. In 14 days, yellow crystals, suitable for data collection, were observed on the sides of the vial.

### Refinement

H atoms were positioned geometrically with C—H = 0.95, 0.96 and 1.00 Å, for aromatic, methyl and methine H atoms, respectively, and constrained to ride on their parent atoms, with *U*_iso_(H) = 1.2Ueq(C)[*U*_iso_(H) = 1.5Ueq(C) for methyl groups].

### Crystal data

**Table:**

C_18_H_12_N_6_O_2_·CHCl_3_	*F*(000) = 944
*M**_r_* = 463.70	*D*_x_ = 1.645 Mg m^−^^3^
Monoclinic, *P*2_1_/*n*	Cu *K*α radiation, λ = 1.54178 Å
Hall symbol: -P 2yn	Cell parameters from 8311 reflections
*a* = 8.9043 (2) Å	θ = 4.3–71.9°
*b* = 16.4009 (4) Å	µ = 4.72 mm^−^^1^
*c* = 13.4872 (4) Å	*T* = 173 K
β = 108.058 (1)°	Transparent prism, yellow
*V* = 1872.63 (8) Å^3^	0.45 × 0.22 × 0.17 mm
*Z* = 4	

### Data collection

**Table:**

Bruker APEXII CCD diffractometer	3370 independent reflections
Radiation source: fine-focus sealed tube	3115 reflections with *I* > 2σ(*I*)
graphite	*R*_int_ = 0.033
phi and ω scans	θ_max_ = 68.2°, θ_min_ = 4.4°
Absorption correction: numerical (*SADABS*; Bruker, 2008)	*h* = −10→10
*T*_min_ = 0.224, *T*_max_ = 0.508	*k* = −19→19
15543 measured reflections	*l* = −16→15

### Refinement

**Table:**

Refinement on *F*^2^	Primary atom site location: structure-invariant direct methods
Least-squares matrix: full	Secondary atom site location: difference Fourier map
*R*[*F*^2^ > 2σ(*F*^2^)] = 0.032	Hydrogen site location: inferred from neighbouring sites
*wR*(*F*^2^) = 0.095	H-atom parameters constrained
*S* = 1.08	*w* = 1/[σ^2^(*F*_o_^2^) + (0.061*P*)^2^ + 0.6946*P*] where *P* = (*F*_o_^2^ + 2*F*_c_^2^)/3
3370 reflections	(Δ/σ)_max_ = 0.001
273 parameters	Δρ_max_ = 0.37 e Å^−^^3^
0 restraints	Δρ_min_ = −0.26 e Å^−^^3^

### Special details

**Table:**

Geometry. All e.s.d.'s (except the e.s.d. in the dihedral angle between two l.s. planes) are estimated using the full covariance matrix. The cell e.s.d.'s are taken into account individually in the estimation of e.s.d.'s in distances, angles and torsion angles; correlations between e.s.d.'s in cell parameters are only used when they are defined by crystal symmetry. An approximate (isotropic) treatment of cell e.s.d.'s is used for estimating e.s.d.'s involving l.s. planes.
Refinement. Refinement of *F*^2^ against ALL reflections. The weighted *R*-factor *wR* and goodness of fit *S* are based on *F*^2^, conventional *R*-factors *R* are based on *F*, with *F* set to zero for negative *F*^2^. The threshold expression of *F*^2^ > σ(*F*^2^) is used only for calculating *R*-factors(gt) *etc*. and is not relevant to the choice of reflections for refinement. *R*-factors based on *F*^2^ are statistically about twice as large as those based on *F*, and *R*- factors based on ALL data will be even larger.

### Fractional atomic coordinates and isotropic or equivalent isotropic displacement parameters (Å^2^)

**Table:**

	*x*	*y*	*z*	*U*_iso_*/*U*_eq_	
Cl1	1.11933 (6)	0.91490 (3)	0.06882 (4)	0.03314 (15)	
Cl2	1.02227 (5)	0.75947 (3)	0.13113 (3)	0.02516 (14)	
Cl3	0.78834 (5)	0.87388 (3)	0.00893 (4)	0.03132 (15)	
O1	0.58359 (16)	0.89641 (8)	0.76117 (10)	0.0252 (3)	
O2	0.50035 (16)	1.16797 (8)	0.78817 (10)	0.0253 (3)	
N1	0.93260 (18)	0.83887 (9)	0.33300 (11)	0.0211 (3)	
N2	0.69578 (17)	0.92402 (9)	0.59325 (11)	0.0185 (3)	
N3	0.53018 (17)	1.03163 (9)	0.76877 (11)	0.0199 (3)	
N4	0.61174 (18)	1.12873 (9)	0.66532 (11)	0.0193 (3)	
N5	0.71061 (17)	1.08699 (9)	0.53254 (11)	0.0185 (3)	
N6	0.94027 (18)	0.99643 (9)	0.27110 (11)	0.0206 (3)	
C1	0.9330 (2)	0.76237 (11)	0.36516 (14)	0.0228 (4)	
H1	0.9744	0.7215	0.3310	0.027*	
C2	0.8759 (2)	0.73847 (11)	0.44641 (15)	0.0232 (4)	
H2	0.8798	0.6829	0.4670	0.028*	
C3	0.8141 (2)	0.79660 (11)	0.49591 (14)	0.0207 (4)	
H3	0.7739	0.7820	0.5510	0.025*	
C4	0.8113 (2)	0.87791 (10)	0.46354 (13)	0.0180 (3)	
C5	0.7528 (2)	0.94334 (10)	0.51497 (13)	0.0181 (3)	
C6	0.6493 (2)	0.98516 (11)	0.64097 (13)	0.0187 (4)	
C7	0.5877 (2)	0.96493 (10)	0.72797 (13)	0.0193 (4)	
C8	0.5445 (2)	1.11330 (11)	0.74324 (13)	0.0202 (4)	
C9	0.6584 (2)	1.06698 (10)	0.61137 (13)	0.0185 (4)	
C10	0.7584 (2)	1.02511 (10)	0.48401 (13)	0.0179 (3)	
C11	0.8194 (2)	1.04465 (11)	0.39814 (13)	0.0186 (3)	
C12	0.8254 (2)	1.12494 (11)	0.36400 (14)	0.0211 (4)	
H12	0.7857	1.1686	0.3949	0.025*	
C13	0.8895 (2)	1.13969 (11)	0.28512 (14)	0.0221 (4)	
H13	0.8958	1.1936	0.2609	0.027*	
C14	0.9452 (2)	1.07352 (11)	0.24152 (14)	0.0226 (4)	
H14	0.9895	1.0843	0.1872	0.027*	
C15	0.8776 (2)	0.98147 (10)	0.34867 (13)	0.0185 (3)	
C16	0.8732 (2)	0.89648 (11)	0.38207 (13)	0.0181 (4)	
C17	0.4656 (2)	1.01675 (11)	0.85485 (14)	0.0241 (4)	
H17A	0.4159	0.9628	0.8465	0.036*	
H17B	0.3867	1.0586	0.8543	0.036*	
H17C	0.5511	1.0189	0.9213	0.036*	
C18	0.6292 (2)	1.21489 (11)	0.64044 (14)	0.0232 (4)	
H18A	0.7078	1.2195	0.6034	0.035*	
H18B	0.6641	1.2466	0.7051	0.035*	
H18C	0.5275	1.2359	0.5962	0.035*	
C19	0.9722 (2)	0.86285 (11)	0.10784 (14)	0.0226 (4)	
H19	0.9645	0.8881	0.1736	0.027*	

### Atomic displacement parameters (Å^2^)

**Table:**

	*U*^11^	*U*^22^	*U*^33^	*U*^12^	*U*^13^	*U*^23^
Cl1	0.0291 (3)	0.0310 (3)	0.0357 (3)	−0.00401 (19)	0.0049 (2)	0.01122 (19)
Cl2	0.0350 (3)	0.0176 (2)	0.0227 (2)	0.00243 (17)	0.00862 (19)	0.00046 (15)
Cl3	0.0251 (3)	0.0264 (3)	0.0357 (3)	0.00431 (18)	−0.0004 (2)	−0.00619 (18)
O1	0.0364 (7)	0.0162 (6)	0.0245 (7)	−0.0019 (5)	0.0115 (6)	−0.0004 (5)
O2	0.0348 (7)	0.0187 (6)	0.0233 (6)	0.0043 (5)	0.0104 (6)	−0.0023 (5)
N1	0.0255 (8)	0.0172 (7)	0.0193 (7)	−0.0007 (6)	0.0048 (6)	−0.0020 (6)
N2	0.0213 (7)	0.0153 (7)	0.0167 (7)	−0.0020 (6)	0.0023 (6)	−0.0017 (5)
N3	0.0240 (7)	0.0175 (7)	0.0179 (7)	−0.0003 (6)	0.0059 (6)	−0.0019 (5)
N4	0.0258 (8)	0.0133 (7)	0.0179 (7)	0.0018 (6)	0.0054 (6)	−0.0010 (5)
N5	0.0213 (7)	0.0146 (7)	0.0170 (7)	0.0009 (5)	0.0023 (6)	0.0001 (5)
N6	0.0255 (8)	0.0180 (7)	0.0168 (7)	−0.0011 (6)	0.0042 (6)	0.0004 (6)
C1	0.0286 (9)	0.0162 (9)	0.0228 (9)	0.0007 (7)	0.0067 (8)	−0.0030 (7)
C2	0.0296 (9)	0.0141 (8)	0.0244 (9)	0.0000 (7)	0.0065 (8)	0.0000 (7)
C3	0.0239 (9)	0.0179 (9)	0.0189 (8)	−0.0021 (7)	0.0047 (7)	−0.0011 (7)
C4	0.0194 (8)	0.0150 (8)	0.0165 (8)	−0.0018 (6)	0.0011 (7)	−0.0015 (6)
C5	0.0194 (8)	0.0161 (8)	0.0159 (8)	−0.0021 (7)	0.0015 (7)	−0.0014 (6)
C6	0.0201 (8)	0.0168 (8)	0.0167 (8)	−0.0007 (7)	0.0019 (7)	−0.0014 (6)
C7	0.0227 (8)	0.0164 (9)	0.0164 (8)	−0.0023 (7)	0.0025 (7)	−0.0026 (6)
C8	0.0221 (9)	0.0183 (8)	0.0172 (8)	0.0007 (7)	0.0018 (7)	−0.0006 (7)
C9	0.0193 (8)	0.0164 (8)	0.0165 (8)	−0.0002 (7)	0.0006 (7)	−0.0021 (6)
C10	0.0184 (8)	0.0161 (8)	0.0163 (8)	−0.0001 (6)	0.0011 (7)	−0.0017 (6)
C11	0.0198 (8)	0.0168 (8)	0.0161 (8)	−0.0002 (7)	0.0012 (7)	0.0006 (6)
C12	0.0228 (9)	0.0172 (9)	0.0198 (9)	0.0009 (7)	0.0015 (7)	−0.0010 (7)
C13	0.0256 (9)	0.0175 (8)	0.0201 (9)	−0.0015 (7)	0.0024 (7)	0.0033 (7)
C14	0.0266 (9)	0.0209 (9)	0.0187 (8)	−0.0027 (7)	0.0047 (7)	0.0017 (7)
C15	0.0206 (8)	0.0163 (9)	0.0154 (8)	−0.0007 (7)	0.0008 (7)	−0.0003 (6)
C16	0.0193 (8)	0.0158 (8)	0.0156 (8)	−0.0006 (7)	0.0000 (7)	−0.0018 (6)
C17	0.0314 (10)	0.0217 (9)	0.0210 (9)	−0.0015 (7)	0.0105 (8)	−0.0015 (7)
C18	0.0339 (10)	0.0139 (8)	0.0218 (9)	0.0014 (7)	0.0084 (8)	−0.0002 (7)
C19	0.0264 (9)	0.0191 (8)	0.0197 (9)	−0.0006 (7)	0.0036 (7)	−0.0005 (7)

### Geometric parameters (Å, °)

**Table:**

Cl1—C19	1.7747 (18)	C3—C4	1.401 (2)
Cl2—C19	1.7564 (18)	C3—H3	0.9500
Cl3—C19	1.7716 (19)	C4—C16	1.407 (2)
O1—C7	1.214 (2)	C4—C5	1.459 (2)
O2—C8	1.214 (2)	C5—C10	1.410 (2)
N1—C1	1.327 (2)	C6—C9	1.410 (2)
N1—C16	1.352 (2)	C6—C7	1.479 (2)
N2—C6	1.325 (2)	C10—C11	1.459 (2)
N2—C5	1.343 (2)	C11—C12	1.401 (2)
N3—C7	1.392 (2)	C11—C15	1.415 (2)
N3—C8	1.399 (2)	C12—C13	1.376 (3)
N3—C17	1.467 (2)	C12—H12	0.9500
N4—C9	1.384 (2)	C13—C14	1.396 (3)
N4—C8	1.384 (2)	C13—H13	0.9500
N4—C18	1.472 (2)	C14—H14	0.9500
N5—C9	1.327 (2)	C15—C16	1.469 (2)
N5—C10	1.346 (2)	C17—H17A	0.9800
N6—C14	1.331 (2)	C17—H17B	0.9800
N6—C15	1.352 (2)	C17—H17C	0.9800
C1—C2	1.399 (3)	C18—H18A	0.9800
C1—H1	0.9500	C18—H18B	0.9800
C2—C3	1.373 (3)	C18—H18C	0.9800
C2—H2	0.9500	C19—H19	1.0000
			
C1—N1—C16	117.55 (15)	N5—C10—C11	118.16 (15)
C6—N2—C5	117.01 (15)	C5—C10—C11	120.02 (15)
C7—N3—C8	125.62 (15)	C12—C11—C15	118.44 (16)
C7—N3—C17	117.70 (15)	C12—C11—C10	121.93 (16)
C8—N3—C17	116.25 (14)	C15—C11—C10	119.62 (15)
C9—N4—C8	122.44 (15)	C13—C12—C11	119.12 (16)
C9—N4—C18	120.82 (14)	C13—C12—H12	120.4
C8—N4—C18	116.73 (14)	C11—C12—H12	120.4
C9—N5—C10	116.45 (15)	C12—C13—C14	118.39 (16)
C14—N6—C15	117.66 (15)	C12—C13—H13	120.8
N1—C1—C2	123.77 (16)	C14—C13—H13	120.8
N1—C1—H1	118.1	N6—C14—C13	124.28 (16)
C2—C1—H1	118.1	N6—C14—H14	117.9
C3—C2—C1	118.95 (17)	C13—C14—H14	117.9
C3—C2—H2	120.5	N6—C15—C11	122.09 (16)
C1—C2—H2	120.5	N6—C15—C16	117.76 (15)
C2—C3—C4	118.70 (16)	C11—C15—C16	120.14 (15)
C2—C3—H3	120.7	N1—C16—C4	122.58 (16)
C4—C3—H3	120.7	N1—C16—C15	117.50 (15)
C3—C4—C16	118.44 (16)	C4—C16—C15	119.91 (15)
C3—C4—C5	121.75 (15)	N3—C17—H17A	109.5
C16—C4—C5	119.76 (16)	N3—C17—H17B	109.5
N2—C5—C10	120.93 (16)	H17A—C17—H17B	109.5
N2—C5—C4	118.54 (15)	N3—C17—H17C	109.5
C10—C5—C4	120.52 (15)	H17A—C17—H17C	109.5
N2—C6—C9	121.92 (16)	H17B—C17—H17C	109.5
N2—C6—C7	117.64 (16)	N4—C18—H18A	109.5
C9—C6—C7	120.43 (16)	N4—C18—H18B	109.5
O1—C7—N3	121.63 (16)	H18A—C18—H18B	109.5
O1—C7—C6	124.15 (16)	N4—C18—H18C	109.5
N3—C7—C6	114.20 (15)	H18A—C18—H18C	109.5
O2—C8—N4	121.82 (16)	H18B—C18—H18C	109.5
O2—C8—N3	121.06 (16)	Cl2—C19—Cl3	110.94 (10)
N4—C8—N3	117.12 (15)	Cl2—C19—Cl1	110.33 (10)
N5—C9—N4	118.51 (16)	Cl3—C19—Cl1	108.81 (10)
N5—C9—C6	121.85 (16)	Cl2—C19—H19	108.9
N4—C9—C6	119.65 (16)	Cl3—C19—H19	108.9
N5—C10—C5	121.81 (16)	Cl1—C19—H19	108.9
			
C16—N1—C1—C2	−0.1 (3)	N2—C6—C9—N5	1.6 (3)
N1—C1—C2—C3	0.7 (3)	C7—C6—C9—N5	−178.85 (16)
C1—C2—C3—C4	−0.4 (3)	N2—C6—C9—N4	−178.51 (16)
C2—C3—C4—C16	−0.5 (3)	C7—C6—C9—N4	1.0 (3)
C2—C3—C4—C5	−177.73 (17)	C9—N5—C10—C5	0.3 (2)
C6—N2—C5—C10	−1.6 (2)	C9—N5—C10—C11	−178.90 (15)
C6—N2—C5—C4	177.43 (15)	N2—C5—C10—N5	1.4 (3)
C3—C4—C5—N2	−2.1 (3)	C4—C5—C10—N5	−177.55 (16)
C16—C4—C5—N2	−179.32 (15)	N2—C5—C10—C11	−179.40 (15)
C3—C4—C5—C10	176.93 (16)	C4—C5—C10—C11	1.6 (3)
C16—C4—C5—C10	−0.3 (3)	N5—C10—C11—C12	−1.6 (3)
C5—N2—C6—C9	0.1 (3)	C5—C10—C11—C12	179.22 (16)
C5—N2—C6—C7	−179.42 (15)	N5—C10—C11—C15	177.03 (16)
C8—N3—C7—O1	173.01 (17)	C5—C10—C11—C15	−2.2 (2)
C17—N3—C7—O1	0.8 (3)	C15—C11—C12—C13	−1.0 (3)
C8—N3—C7—C6	−8.3 (2)	C10—C11—C12—C13	177.63 (16)
C17—N3—C7—C6	179.50 (15)	C11—C12—C13—C14	0.5 (3)
N2—C6—C7—O1	3.3 (3)	C15—N6—C14—C13	−0.2 (3)
C9—C6—C7—O1	−176.22 (17)	C12—C13—C14—N6	0.1 (3)
N2—C6—C7—N3	−175.31 (15)	C14—N6—C15—C11	−0.3 (3)
C9—C6—C7—N3	5.1 (2)	C14—N6—C15—C16	−179.49 (15)
C9—N4—C8—O2	−178.08 (16)	C12—C11—C15—N6	1.0 (3)
C18—N4—C8—O2	0.8 (3)	C10—C11—C15—N6	−177.72 (16)
C9—N4—C8—N3	2.3 (3)	C12—C11—C15—C16	−179.93 (16)
C18—N4—C8—N3	−178.82 (15)	C10—C11—C15—C16	1.4 (2)
C7—N3—C8—O2	−174.77 (17)	C1—N1—C16—C4	−0.8 (3)
C17—N3—C8—O2	−2.5 (2)	C1—N1—C16—C15	178.08 (16)
C7—N3—C8—N4	4.9 (3)	C3—C4—C16—N1	1.1 (3)
C17—N3—C8—N4	177.20 (15)	C5—C4—C16—N1	178.42 (15)
C10—N5—C9—N4	178.39 (15)	C3—C4—C16—C15	−177.78 (16)
C10—N5—C9—C6	−1.7 (2)	C5—C4—C16—C15	−0.5 (3)
C8—N4—C9—N5	174.92 (15)	N6—C15—C16—N1	0.1 (2)
C18—N4—C9—N5	−4.0 (2)	C11—C15—C16—N1	−179.05 (15)
C8—N4—C9—C6	−5.0 (3)	N6—C15—C16—C4	179.05 (15)
C18—N4—C9—C6	176.14 (16)	C11—C15—C16—C4	−0.1 (3)

### Hydrogen-bond geometry (Å, °)

**Table:**

*D*—H···*A*	*D*—H	H···*A*	*D*···*A*	*D*—H···*A*
C19—H19···N1	1.00	2.39	3.188 (2)	136.
C19—H19···N6	1.00	2.26	3.181 (2)	152.
